# Effect of Environmental Chemical Stress on Nuclear Noncoding RNA Involved in Epigenetic Control

**DOI:** 10.1155/2015/761703

**Published:** 2015-08-03

**Authors:** Patrizio Arrigo, Alessandra Pulliero

**Affiliations:** ^1^National Research Council (CNR), Institute for Macromolecular Studies (ISMAC), Via De Marini 6, 16149 Genoa, Italy; ^2^Department of Health Sciences, University of Genoa, Via A. Pastore 1, 16132 Genoa, Italy

## Abstract

In the last decade the role of noncoding RNAs (ncRNAs) emerges not only as key elements of posttranscriptional gene silencing, but also as important players of epigenetic regulation. New kind and new functions of ncRNAs are continuously discovered and one of their most important roles is the mediation of environmental signals, both physical and chemical. The activity of cytoplasmic short ncRNA is extensively studied, in spite of the fact that their function and role in the nuclear compartment are not yet completely unraveled. Cellular nucleus contains a multiplicity of long and short ncRNAs controlling at different levels transcriptional and epigenetic processes. In addition, some ncRNAs are involved in RNA editing and quality control. In this paper we review the existing knowledge dealing with how chemical stressors can influence the functionality of short nuclear ncRNAs. Furthermore, we perform bioinformatics analyses indicating that chemical environmental stressors not only induce DNA damage but also influence the mechanism of ncRNAs production and control.

## 1. Introduction

The term “epigenetic” was originally used in the field of evolution and development [1]. The advancement of knowledge about molecular mechanisms that control the gene expression has profoundly changed the meaning of the term, which can assume different facets that are based on the field of interest of a researcher. Recent studies shed light on the role of environment in epigenetic processes and, as a consequence, a new definition of epigenetic has been proposed [2]. This interpretation considers the epigenetic as structural changes, at chromosome level, that allow sensing, storing, and transmitting modifications of biological activity. This proposed definition considers the modification of chromosomal marks associated with genomic and cellular changes. The proposed definition does not take into account other events, both endogenous (structural) and exogenous, except when they affect chromosome functionality. Probably epigenetic mechanisms operate as a buffer for genetic variations that are waiting to change the expression of genes leading to a different molecular phenotype.

The next-generation sequencing and other high throughput methods have underlined the relevance of epigenetic process in cellular homeostasis. A large number of experiments have demonstrated epigenetic alteration in several pathological processes [3]. It is important to note the existence of a feedback between metabolic network and epigenetic activity [4, 5]. The knowledge about the mechanisms connecting metabolic network and epigenetic is even now to be deeply investigated. The goal of epigenetic is the transcriptional control by several different mechanisms. The fundamental elements are (a) the DNA methylation; (b) the histone modification; (c) nucleosomal positioning; and (d) noncoding RNAs. DNA methylation, histone modifications, and nucleosomal positioning are the mechanism studied for a long time; however only more recently the role of noncoding RNAs (ncRNAs) in epigenetic processes has been highlighted. The results of deep sequencing project, such as ENCODE, have pointed out a greater than expected transcriptional activity of genome. These high throughput experiments have also screened an amount of expressed ncRNAs larger than protein-coding genes [6]. Systematic bioinformatics analysis has supported the investigation of relation between ncRNAs and epigenetic mechanisms [7]. The advancement of experimental activity has unrevealed the complexity of ncRNA world. Nowadays it is known that ncRNAs are involved in processes such as transcription, translation, RNA editing, and protection against exogenous nucleic acids. The role of ncRNAs in the response against the environmental stress has initially been identified in bacteria [8], subsequently in plant [9], and then in animals. The length of ncRNAs is a discriminating feature and, even if the initial focus of research has been the class of short ncRNAs, the long noncoding RNAs have acquired a considerable significance in the exploration of epigenetic mechanisms.

It is difficult to investigate how each type of ncRNAs cooperates in the epigenetic control and to examine the hierarchy and timing of their control activity. The aim of this paper is to give a general framework of the overall epigenetic control regulated by noncoding RNA taking their structural characteristics into account. In particular, we would like to highlight those elements that are predominantly involved in the response against environmental stress.

## 2. The Noncoding RNA World

It has been commonly accepted that ncRNAs were not functional and were therefore labeled as “junk RNAs.” At the same time, the function of the noncoding transcripts and their relevance to diseases have remained undefined.

Many of the putative functional ncRNAs are present at very low levels and thus unlikely to be of any importance with respect to cell or organism physiology. Additionally, the abundance of an ncRNA species shortly correlates with its level of conservation [10], which is a good agent for function [11, 12], thus determining the relative abundance of a given ncRNA in the relevant cell type. The majority of human cellular RNA consists of rRNA (~90% of total RNA). Total number of RNA molecules is estimated to be about 10^7^ per cell, and ncRNAs include snRNA, snoRNA, and miRNA. Although there is less tRNA by mass, ncRNAs small size results in their molar level being higher than rRNA [13]. Other abundant RNAs, such as mRNAs, snRNAs, and snoRNAs, are present in aggregate at levels that are about 1-2 orders of magnitude lower than rRNA and tRNA. Certain small RNAs, such as miRNAs and piRNAs, can be present at very high levels; however, this appears to be cell type dependent [11]. Moreover, the overexpression of lncRNAs can potentially measure the cytotoxicity signals of various environmental stresses. Indeed, some researchers have developed human cells transfected with lncRNAs used as a sensor of cytotoxicity for a broad range of environmental stresses. They identified three lncRNAs (GAS5, IDI2-AS1, and SNHG15) that respond to several environmental stresses and found overexpression of these lncRNAs sensitized human cells to cell death by the stresses. These sensor cells with overexpressed lncRNAs can potentially report cytotoxicity signals of various environmental stresses [14].

This explosion can be related with the increased number of biological functionalities operated by these molecules. In spite of the simple initial classification based on their length, a new more refined categorization has been proposed [15]. Volders et al. describe a novel database for human long noncoding RNA, constituted by a large and diverse class of noncoding RNA genes. LNCipedia offers 21 488 human lncRNA transcripts obtained from different sources. They underline that, much like microRNAs, many lncRNAs have a significant secondary structure, in line with their presumed association with proteins or protein complexes [16].

According to this rule we can classify them into “nuclear” and “cytoplasmic” noncoding RNAs. It is important to clarify that even if the ncRNA is produced in the cytoplasm but acts in the nucleus it is considered nuclear. The proposed approach allows better identifying those classes that can influence epigenetic mechanisms and RNA processing and also having more valuable information about the differential effect of environmental stressors on the two cellular compartments. Knowing the location of such ncRNAs could help in selecting the best candidates for starting the ncRNA-based gene therapy trials. For example, the fact that miRNA overexpression in cancer cells has a pathogenic effect provides the rationale for using miRNAs as potential therapeutic targets in cancer.

Cytoplasmic compartment could also be influenced by noncoding RNA originated far from one tissue (circulating ncRNAs) or by exogenous ncRNAs (viral miRNAs). In an epigenetic perspective the role of ncRNA in the nucleus bears particular interest. Noncoding RNAs act in nucleus as long ncRNAs and in nucleolus as short ncRNAs.

The long ncRNA (lncRNA) action affects directly the transcriptional process. The short ncRNA acting in the nucleolus is principally entailed in RNA quality control. It is crucial to recall that ncRNAs are originated by a transcriptional process comparable with those regulating protein transcription. The localization of genomic regions encoding ncRNA is not yet completely clarified. In terms of ncRNA transcription, it is known they can be originated near the locus that they regulate (*cis*-regulation) or distally to their target (*trans*-regulation). In the paragraph below we summarize the functional differences between strictly nuclear ncRNAs (e.g., lncRNAs, short ncRNAs such as piRNAs, paRNAs, and rasiRNAs) and nucleolar RNAs. In order to better correlate their structural and functional characteristics, we separately analyzed the main groups of nuclear and nucleolar ncRNAs. This approach is valuable to understand ncRNA cooperative effects on epigenetic regulation. In particular our interest is to describe the functional role of those ncRNAs operating in nuclear environment because, leaving out the important exception of mitochondria, the epigenetic control occurs in the nucleus. This survey also attempts to find common elements that help to analyze the DNA damage and repair by a different integrative perspective. We guess that clarifying the functional feedback between different noncoding regulatory RNAs helps to decipher those steps that are more sensitive to induced damage by environmental factors in the perspective to assess the long term risk for human health.

## 3. The Nuclear Small ncRNAs (snRNAs)

The nuclear activity regulation by RNA is a dynamic process in which are involved ncRNAs originated from nuclear compartment and some of them are processed in the cytoplasm. The regulatory ncRNAs, according to their length, can be divided into small ncRNAs (sncRNAs, smaller than 200 nucleotides (nts)) and long ncRNAs (lncRNAs, longer than 200 nts). There are also ncRNAs with the length of 60–300 nts, called small nucleolar RNAs (snoRNAs). The small ncRNAs include Piwi-associated RNAs (piRNAs), microRNAs (miRNAs), small interfering RNAs (siRNAs), transcription initiation RNAs (tiRNAs), and other small ncRNAs [17].

The short nuclear RNAs (snRNAs) are classified in two different groups according to the proteins they interact with [18]. The first group is identified as “Sm-class” because they interact with Sm proteins. The second group is called “Lsm-class” according to the name of their interacting proteins. These proteins and snRNA cooperate in spliceosome formation [19]. Conversely, it is important to underline the transcriptional differences between the two classes. The “Sm-class” is transcribed by Pol II while the “Lsm-class” is transcribed by Pol III.

**Figure 1:**
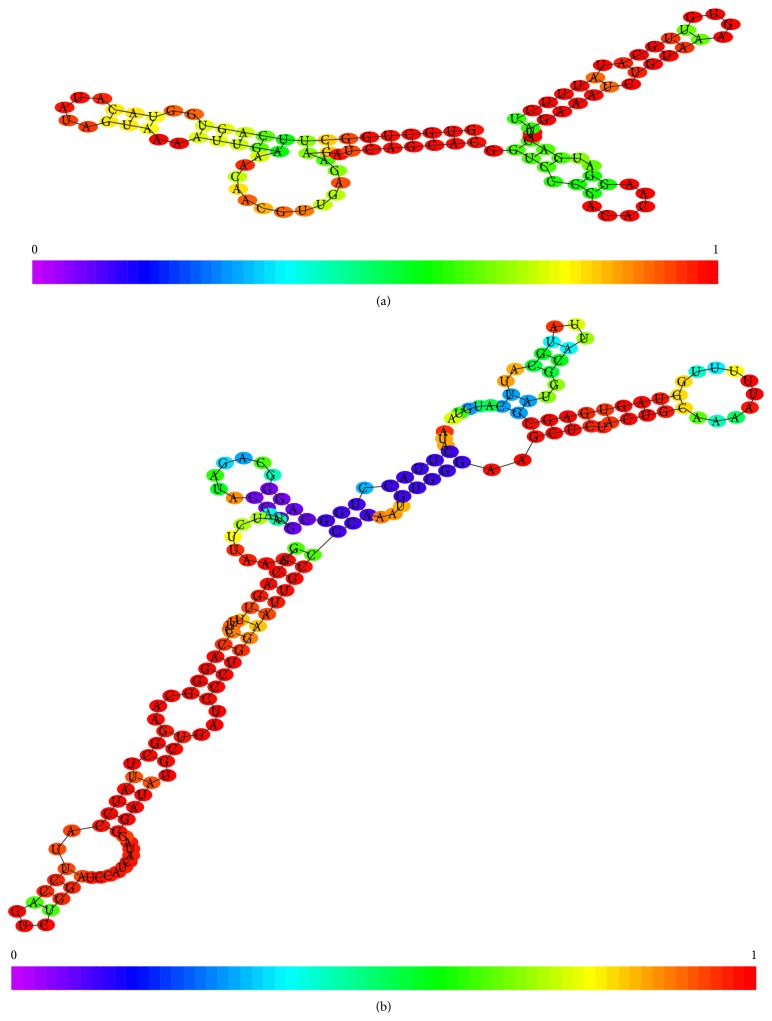
(a) 2D structure of U6 snRNA (Lsm-class). (b) 2D structure of U1 snRNA (Sm-class).

Another important discriminative element between these two types of snRNAs is their biogenetic pathway. The “Sm-class” requires a cytoplasmic phase, in spite of the fact that “Lsm-class” biogenesis is exclusively completed in the nucleus. The two groups show structural differences as highlighted in Figure 1. From the structural point of view, the 5′ end is discriminative between Sm-class and Lsm-class. In the first group there is a trimethylguanosine, while the second group of ncRNAs only have one methyl group in the 5′ cap. The protein selectivity is driven by the presence of specific nucleotide motifs. In the “Sm-class” the recognition motif is located between two regular stem loops and it has the following composition: AUUUGUG (Sm site) and GAAGCUG (Lsm site) [20].

**Figure 2:**
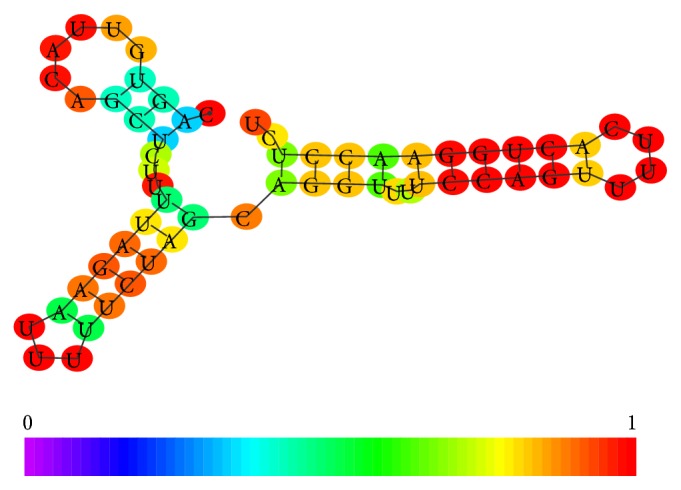
Conformation of Human U7 cDNA (ENST00000459276).

Among these small snRNAs the U7 seems to have a role in epigenetic control by repression of histone genes [21]; its structure is shown in Figure 2. It differs from other Sm snRNAs because it recruits a different set of Sm proteins.

In addition to this specific nuclear small noncoding RNA other ribonucleotide oligomers have function in epigenetic control. In particular we consider the Piwi-interacting RNAs (piRNAs), the promoter-associated RNAs (paRNAs), and the repeat associated RNAs (rasiRNAs).

Piwi-interacting RNAs (piRNAs) are a novel class of sncRNAs, with a length of 26–31 nts, which specifically interact with P-element-induced wimpy testis (Piwi) protein in the Argonaute group of proteins. Piwi proteins in humans have four homologs: PiwiL1/Hiwi, PiwiL2/Hili, PiwiL3, and PiwiL4/Hiwi2 [22]. Studies have reported that Hiwi may take part in germ cell proliferation and carcinogenesis process [23].

The piRNAs are short noncoding RNAs little bit longer than miRNAs; they do not show a significant sequence conservation. They, at variance with miRNAs and siRNAs, are functionalized by a DICER independent pathway. They interact with the AGO3, a member of Argonaute family that is peculiar of germ line cells [24].

piRNAs are found in germ line cells, especially in mammals; for example, several million piRNAs are found in mammalian testes. Genetic regions that encode piRNAs consist of clusters. These clusters have repeats of piRNA sequences and there can be as many as 1000 copies of piRNAs in a cluster. piRNAs are processed from long precursors transcripts but little is known of the biogenesis of piRNAs. Some piRNA clusters consist of transposon sequences. A major role of the piRNA/Piwi protein complex in germ line cells is to protect cells from invading transposons. When the cell encounters a transposon that it has not been exposed to before, the transposable elements (TE) by chance may incorporate into the DNA in a piRNA-encoding cluster and thus its sequence can become part of the piRNA cluster. This is a type of “genetic immune system” that is found in both eukaryotes and prokaryotes. For example, the CRISPR complex in bacteria has a mechanism to protect cells from invasion by plasmids and viruses [25]. The piRNA/Piwi complex is also essential in genetic imprinting in the case involving DNA methylation of the imprinted locus Ras protein-specific guanine nucleotide-releasing factor 1 locus in mouse germ line cells [26]. In nematodes, piRNAs detect a TE sequence via imperfect base-pairing and then induce another small RNA class, termed 22G-RNAs to silence a transposon [27]. Some processes involve epigenetic mechanisms. For example, in* Drosophila*, nuclear piRNAs can target a transposon and direct Piwi proteins repress chromatin and transcription of the TE [28]. Additionally, piRNAs may also induce the methylation of TE LINE-1 DNA in humans.

The primary task of piRNAs is to preserve the genome integrity by repression TE. The transposon repositioning can induce some genomic damage in germ line. It is interesting to underline that, at variance with the precursors of other short ncRNAs such as miRNA or snRNA, the precursors of piRNA are vesiculated in cytoplasm without a shuttle complex like RanGTp-exportin 5 required by pre-miRNA. piRNA maturation and Piwi-piRNA complex (Piwi-piRISC) formation occur in the cytoplasm. Piwi-piRISCs are then imported into the nucleus where they repress TEs at transcriptional level by directing specific histone modifications to TE loci. The piRNAs originate from intergenic repetitive elements that are in many cases clustered. Currently, available knowledge suggests a possible involvement of piRNA in “de novo” methylation of DNA in transposable elements. The piRNAs are indicated as pivotal element in the process of genomic imprinting. It is important to underline that also these small ncRNAs are prone to be methylated [29]. Their stability is affected by methylation because it protects the piRNAs, and other small ncRNAs, from other kinds of modifications, such as uridylation, that determine the speed of RNA degradation.

**Figure 3:**
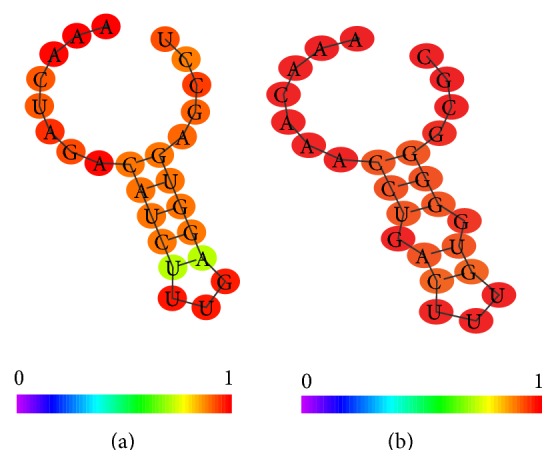
(a) Conformational analysis of a piRNA (NCBI code DQ569927 coding strand minus chromosome 11), (b) conformational analysis of a piRNA (NCBI code DQ569913 coding strand plus chromosome 21).

The enzyme responsible for piRNA methylation is the HEN1, that is, a methyltransferase that methylates miRNAs and siRNAs on the ribose of the last nucleotide. This enzyme introduces a 2′-O-methyl group at 3′ end of a small RNA, including piRNAs. This stabilization process is a critical step to protect the genome integrity as demonstrated by experiments carried out on protozoa [30]. Docking experiments with HEN1 and synthetic short RNA oligomers have suggested, as possible recognition site, a tetramer such as NCGN; these results confirm the well established knowledge about the centrality of CG dinucleotide in methylation process [31]. In Figure 3 piRNA 2D structure and folding are shown.

The repeat associated siRNAs (rasiRNAs) can be considered as subclass of piRNAs. They look like shorter than piRNAs, although their length depends on specific organism. They are originated from an antisense transcript, in spite of the fact that endo-siRNA and miRNA and their fictionalization appear to be DICER1 independent [32]. At the moment the rasiRNAs were identified in low eukaryotes. In order to focus our survey on human we mention the class of small nuclear ncRNA but we do not have enough evidences about their role in human epigenetic control.

## 4. The Nucleolar Small Noncoding RNA

**Figure 4:**
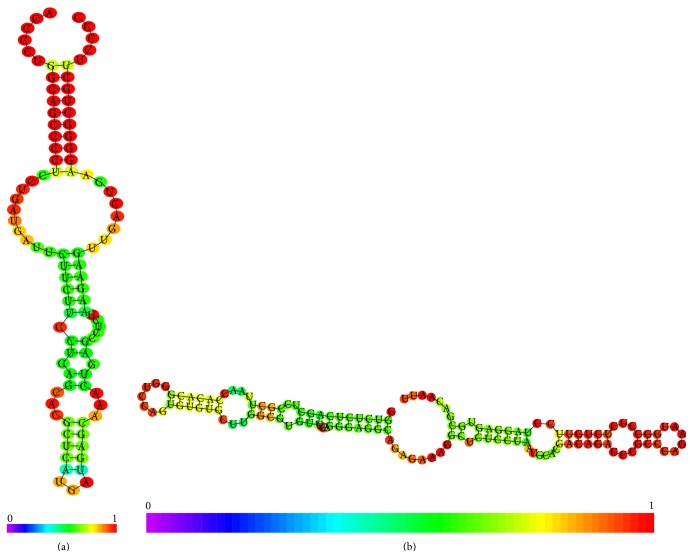
(a) C/D box RNA (SNORD125 NCBI code AM13037.1), (b) H/ACA box RNA (ACA10 NCBI code AJ609432.1).

The nucleolus is the major compartment of the nucleus and it is the place where rRNAs are synthesized. A nuclear compartment can contain more than one nucleolus, that is, surrounded by condensed chromatin layer. In the nucleus there are other organized elements such as Cajal bodies. The regulation of nucleolus assembly and function is primarily committed to specific ncRNAs called small nucleolar RNAs (snoRNAs). Their primary function is to modify other RNAs, not only rRNA but also tRNA and other ncRNAs. These short ribonucleic acids, the most abundant group of intron-encoded ncRNAs, are classified, according to their specific conformational properties, into two main groups: the C/D box and the H/ACA box RNAs. Each group executes a specific modification reaction: the C/D box RNAs operate a RNA methylation reaction, whereas H/ACA box RNAs trigger the change of Uridine into pseudouridine.  Figure 4 shows the architectural differences between C/D box and H/ACA box RNAs. The C/D box is characterized by two nucleotide motifs: the “C box” (RUGAUGA) and the “D box” (CUGA), respectively, located in the 5′ and 3′ ends of the sequence.

The H/ACA snoRNAs have the following canonical motifs: the “H motif” (AgAnnA) located between two conserved stem loops and the box “ACA” near to the 3′ end.

Figure 4 underlines the conformational differences between the two classes of snoRNAs, in particular in the terminal loop that in the C/D box is smaller than in the H/ACA box. The differences in the two ends and in the symmetry of internal bulges are also noticeable. All these features contribute to specifying the regulatory function of these snoRNAs. The two conformations underline the small but significant differences between the piRNA located on the strand plus and the piRNA located on the strand minus. It is interesting to note that only the piRNA located on the strand plus shows a C in the 3′ end. This suggests that this oligomer can be prone to stabilization by methylation. The main distinctive feature is the presence of a small symmetric bulge in the piRNA located on the minus strand.

**Figure 5:**
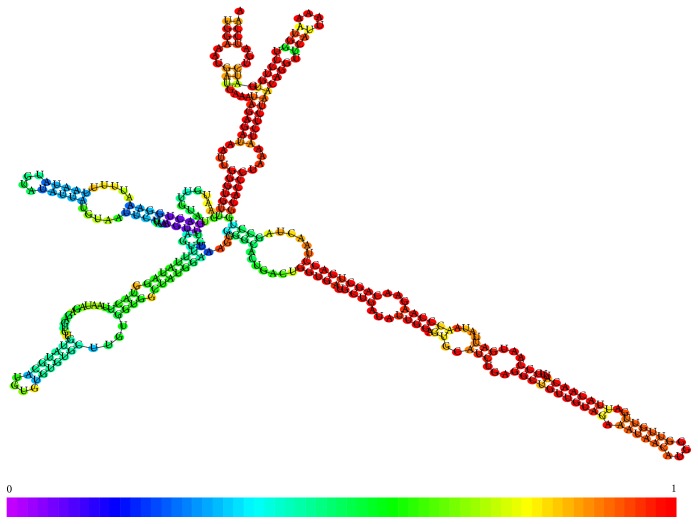
Example of scaRNA conformation (SCANRNA7 NCBI code AY077740.1).

Another ncRNA class containing both types of recognition sites are scaRNA, which are longer than the previously described types. They are associated with the Cajal body, another organized nuclear structure in which also RNPs are processed. Cajal bodies have role in epigenetic control because in these structure the mRNAs encoding histones are processed by snRNAs [33]. The scaRNAs unveil a complex conformational structure that combines both nucleotide feature of C/D and H/ACA box RNAs. Their complexity is exemplified in Figure 5.

The scaRNAs include a specific nucleotide motif UGAG, the Cajal body box (CAB), in the H/ACA domain of the ncRNA [34].

## 5. Long Noncoding RNAs

Long noncoding RNAs (lncRNAs) are transcripts greater than 200 nucleotides in length with little or no protein-coding capability [35]. This arbitrary size threshold distinguishes lncRNAs from other distinct classes of small RNAs such as microRNAs, tRNAs, and snoRNAs. A recent definition of long ncRNA has established a new minimal length of 1000 bp [36].

In the last few years the functional explorations of individual lncRNAs have seen rapid growth. Concomitant with this increased growth of characterized lncRNAs is an increasing understanding toward biological mechanisms, as well as a growing awareness and recognition of the importance of lncRNAs in virtually every cellular and regulatory process [37].

Short ncRNAs are mainly devoted to inducing relative small changes. Conversely, long ncRNAs are capable of directly affecting the transcriptional process mainly by chromatin remodeling [38]. The long ncRNAs are confined in the nucleus and the experimental findings have confirmed their role in transcriptional control by recruitment of proteins responsible for chromatin remodeling. The long ncRNAs interact with promoter of silenced genes but they can also target other distal transcriptional regulatory regions (enhancer or LCR). Indeed, lncRNA-p21 is induced by DNA damage caused by doxorubicin and plays a key role in the p53 transcriptional response [39]. This lncRNA represses p53-regulated genes through binding to heterogeneous nuclear ribonucleoprotein K and modulating its localization, which is necessary for the p53-dependent apoptotic response to DNA damage. Moreover, cycloheximide and hydrogen peroxide dramatically induced these lncRNAs and respond to cellular stresses. Distinct sets of lncRNAs play roles in cellular defense mechanisms against specific stresses, and particular lncRNAs have the potential to be surrogate indicators for cellular stress responses in human-induced pluripotent stem cells [40]. Another study identifies six long ncRNAs (MIR22HG, GABPB-AS1, LINC00152, IDI2-AS1, SNHG15, and FLJ33630) that responded to chemical stressors (cisplatin, cycloheximide, and mercury (II) oxide) in HeLa Tet-off cells. The results indicate that long ncRNAs respond to general and specific chemical stressors. The expression levels of the long ncRNAs were elevated because of prolonged decay rates in response to chemical stressors [41].

## 6. Environmental Stress and Small Nuclear ncRNA and In Silico Test

The above reported sections have summarized the nuclear ncRNA characteristics, functions, and their role in epigenetic control. The knowledge about the damage induced by environmental agents at epigenetic level is well established principally at DNA level, in spite of the fact that the role of noncoding RNAs has become visible more recently. The initial evidence of the ncRNA's role in stress response was obtained by experiments in plants [42] and then in animal models in higher vertebrate and in humans remains to be done. It is necessary to distinguish between an acute and a prolonged environmental stress, this second analytical perspective being valuable in terms of risk assessment and regulatory activity. This survey describes the effect of environmental stress on short snRNAs taking their characteristic conformational features into account. It is important to underline the functional feedback between the cytoplasmic phase of posttranscriptional gene regulation and nuclear epigenetic control. We guess that, in an epigenetic point of view, nuclear compartment seems to be more interesting because also the cytoplasmic ncRNAs such as miRNAs are transcribed in the nucleus and consequently they are also subject to modification induced by snRNAs. In a previous paper [43] we have investigated, by an “in silico” analysis of interaction between a selected sample of chemical environmental pollutant and DICER, a pivotal enzyme of RISC complex. In the present survey, in order to exemplify the possible effects of environmental chemical stress on epigenetic regulation, we have estimated the interaction between three chemical mutagens and some available structures of ncRNAs. These structures represent specific domains of noncoding RNA because to obtain a complete structure is very difficult. In addition some of them are obtained from yeast but, in this case, the evolutionary conservation ensures a quite reliable estimation of interaction.  Table 1 summarizes these results. The 3D structures were retrieved from Nucleic Acid Database (http://ndbserver.rutgers.edu/) and the chemical compound for Pubchem (https://pubchem.ncbi.nlm.nih.gov/). We have used the same docking system PatchDock [44]. The table underlines the type of RNA for each structure. We have used a similar approach using solved 3D structure of snRNA and some synthetic oligomers rather than embedding nucleotide motifs mimicking the natural functional ones such as C box or H box characterizing the snRNAs.  Table 1 summarizes the set of ncRNA structures, the site of interaction, in spite of the protein-nucleic acid interaction; this kind of analysis does not allow estimating the binding energy but only the atomic contact energy and the number of atomic contacts.

**Figure 6:**
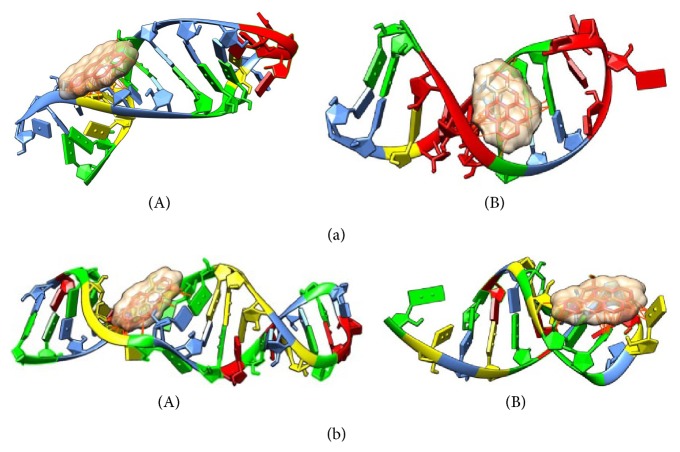
(a) Comparison of complexes between Benzo(a)pyrene and RNA. (A) Complex with a stem loop of human histone mRNA (PDB 1KK); (B) complex with stem loop I of* S. cerevisiae* stem loop I of U2 snRNA (PDB 2O33). Each nucleotide is identified by a different color: A = yellow; C = light blue; G = green; U = red. (b) Comparison of complexes between Benzo(a)pyrene and RNA. (A) Complex with 3′ hairpin of human U65 snRNAs (PDB 2EUY); (B) complex with 3′ stem loop of human U4 snRNA. Each nucleotide is identified by a different color: A = yellow; C = light blue; G = green; U = red.

Table 1 summarizes demonstrative test about the effects of chemical contaminants on small nuclear noncoding RNAs. It is important to underline some interesting considerations: first of all the complex between two small nuclear ncRNAs (U6 and U2) shows the minimal value of estimated contact energy if compared with the other structures. The second interesting finding is the variation of estimated parameter in the 3′ hairpin loop of two different H/ACA snoRNAs and a similar behaviour was pointed out in the two stem loops of U2 (stem loop I) and U6 snRNA. Both these local conformations are located near to 5′ terminal. The last interesting result is the clearly different estimated ACE and contact number in the histone mRNA hairpin. Figures 6(a) and 6(b) illustrate how a chemical contaminant could influence the snRNA functionality by interaction with its conformation.

## 7. Conclusions

Even if the knowledge about epigenetic mechanisms is well established, the new high throughput techniques have disclosed new research perspective. The complexity of RNA world does not yet permit unrevealing the intricate network of feedbacks among different kinds of ncRNAs. The nuclear transcriptional control by nCRNA is pivotal not only for protein expression but also for the production of other ncRNAs. The epigenetic damage, induced by chemical and physical agents, on DNA is well established, in spite of the fact that their effects on the different processes in which ncRNAs are involved are not yet completely investigated. Indeed, the experimental studies are principally focused on the fold change of ncRNAs but precise details of ncRNAs related to structural damage by chemicals are unknown. In this paper we have attempted to demonstrate how a chemical mutagen can trigger damage not only at DNA but also at RNA level. Despite the lack of enough structural information about snoRNAs and the limited set of tested compounds, it is interesting to underline how a chemical mutagen can interfere with the conformation of specific regulatory domains in ncRNAs. It is interesting to note how the chemical entity could induce a slight change in the conformation that could be critical for molecular recognition mainly in the process of RNA editing. Our demonstrative analysis has also pointed out the chemical sensitivity of a hairpin placed into a histone mRNA. It was not possible, for lack of structural information, to analyze the effect of snRNA directly involved in transcriptional process such as promoter-associated, Piwi-interacting, and long noncoding RNAs. We guess that environmental contaminants have the potentiality to modify both levels of genetic information processes.

## Figures and Tables

**Table 1:** Interaction between three different chemical mutagens and some available structures of ncRNAs.

PDB code	Type of RNA	Benzo(a)pyrene		2-Nitrofluorene		4-Nitrosomorpholine	
		CID 2336		CID 11 831		CID60 46	
		ACE	Contacts	ACE	Contacts	ACE	Contacts
2PCW	rRNA bound with H/ACA *ψ* domain	−274.04	57	−231.89	61	−110.60	24

2EUY	U65 H/ACA	−281.06	70	−237.26	55	−113.09	37
	3′ hairpin						
	loop						

2QH3	U64 H/ACA	−279.60	90	−219.53	60	−109.68	31
	3′ hairpin loop						

1MFJ	U4 snRNA	−266.35	76	−218.45	56	−109.02	35
	3′ stem loop						

2LK3	U2/U6 snRNA complex	−224.25	48	−235.16	48	−108.79	21

2LX1	Internal loop	−253.01	79	−189.51	55	−98.88	34

2O33	U2 stem I	−283.93	58	−223.18	78	−117.37	30

1LC6	U6 stem loop	−273.62	65	−226.94	69	−129.48	19

1KKS	Histone mRNA hairpin	−250.27	162	−230.73	165	−133.11	51
